# Mr. Gerald Newton Golden

**Published:** 1991-03

**Authors:** 


### Gerald Newton Golden 1900–1990

We, in the South West have hardly noticed the passing of a distinguished retired orthopaedic surgeon who settled in our midst in 1965. His career was unusual, courageous and very varied. The quiet village of Colyton now became his home but tragically be became a widower within a few years. His career had started abroad in 1923 and he therefore returned to work developing countries, Nigeria, India and more recently Afghanistan, his age he always concealed until it was revealed on his 90th birthday in 1990.

Qualifying in 1923, he left for work in mission hospitals in Brazil where he stayed for the next 5 years. He and another mission doctor proposed including private patients in their care in order to augment the funds for missionary work but the authorities would not agree and he moved to other work in Brazil. He was involved in a typhoid epidemic and was awarded the M.B. by the University of Rio de Janeiro in 1929 for his scientific work in this period.

Returning to Britain in 1929, he gained the FRCS Eng. in 1931 and worked initially in Haslemere as a G.P. surgeon, gaining knowledge and experience so that he was at one time Orthopaedic and Accident Surgeon to the Royal Surrey County Hospital in Guildford, to the Royal Hants County Hospital in Winchester and Orthopaedic surgeon to the Haslemere and District Hospital. He was a very keen Christian throughout his life, clean thinking, resolute and a very kind man. Ill health has afflicted him in recent years, mainly stenocis of his coronary arteries. After his last illness, he insisted on returning to living on his own in Colyton. He persuaded his son to assist him in leaving by air for India where he stayed with former colleagues. He then moved to Kabul where he again became ill and died on 13 October 1990. He would have wished to die in the Developing World, with those to whom he had given so much of his life.

A.L.E-B.

